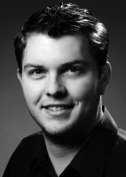# Prey for the Proteasome: Targeted Protein Degradation—A Medicinal Chemist's Perspective

**DOI:** 10.1002/anie.202004310

**Published:** 2020-07-30

**Authors:** Laura M. Luh, Ulrike Scheib, Katrin Juenemann, Lars Wortmann, Michael Brands, Philipp M. Cromm

**Affiliations:** ^1^ Research and Development Pharmaceuticals Bayer AG 13353 Berlin Germany

**Keywords:** chemical biology, medicinal chemistry, molecular glue, PROTACs, targeted protein degradation

## Abstract

Targeted protein degradation (TPD), the ability to control a proteins fate by triggering its degradation in a highly selective and effective manner, has created tremendous excitement in chemical biology and drug discovery within the past decades. The TPD field is spearheaded by small molecule induced protein degradation with molecular glues and proteolysis targeting chimeras (PROTACs) paving the way to expand the druggable space and to create a new paradigm in drug discovery. However, besides the therapeutic angle of TPD a plethora of novel techniques to modulate and control protein levels have been developed. This enables chemical biologists to better understand protein function and to discover and verify new therapeutic targets. This Review gives a comprehensive overview of chemical biology techniques inducing TPD. It explains the strengths and weaknesses of these methods in the context of drug discovery and discusses their future potential from a medicinal chemist's perspective.

## Graphical abstract

**Targeted protein degradation** has created tremendous excitement in chemical biology and drug discovery. This Review gives a comprehensive overview of chemical biology tools as well as therapeutic approaches inducing targeted protein degradation and discusses them from a medicinal chemist's perspective.

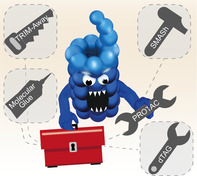

## 1 Introduction

Classical approaches in the drug discovery process typically aim to identify high affinity small molecules modulating the activity of target proteins. This happens in an occupancy‐driven manner with the inhibitor binding to its target and occupying the binding site, thereby inhibiting target function.^1^ This strategy has been very successful for many targets harboring a tractable active or allosteric site like enzymes or receptors. However, the majority of proteins still remains challenging to address, rendering 80–85 % of the human proteome undruggable due to the absence of available binding pockets and suitable chemical matter.^2^ Targeted protein degradation (TPD) offers a novel therapeutic alternative by inducing the depletion or reduction of a disease‐causing protein via hijacking the endogenous protein degradation machineries. TPD has the potential to target the undruggable proteome that limits current drug discovery efforts, as only a binder is required to recruit the target protein for degradation rather than high affinity inhibitors.^3^ In addition to their therapeutic potential, protein degraders are valuable chemical biology tools to validate targets and to gain a deeper understanding of protein function and cellular pathways. An advantage of targeted protein degradation is the acute and rapid depletion of target proteins avoiding unwanted adaption events in comparison to RNA or genome editing tools and the ability to deplete proteins with a slow turnover rate. In contrast to nucleotide‐based methods like RNAi, antisense oligonucleotides or genome editing strategies which all suffer from limited therapeutic applications due to their low in vivo stability and bioavailability,^4^ TPD harnesses more drug‐like small molecules. These degrader molecules are typically inducing novel protein–protein interactions (PPIs) and can either comprise hetero‐bifunctional molecules such as proteolysis‐targeting chimeras (PROTACs)^3^, ^5^ or monovalent molecular glues^6^ which are of non‐chimeric nature. This unique mode of action combining target engagement with subsequent degradation allows them to address targets previously out of reach.

**Figure 1:**
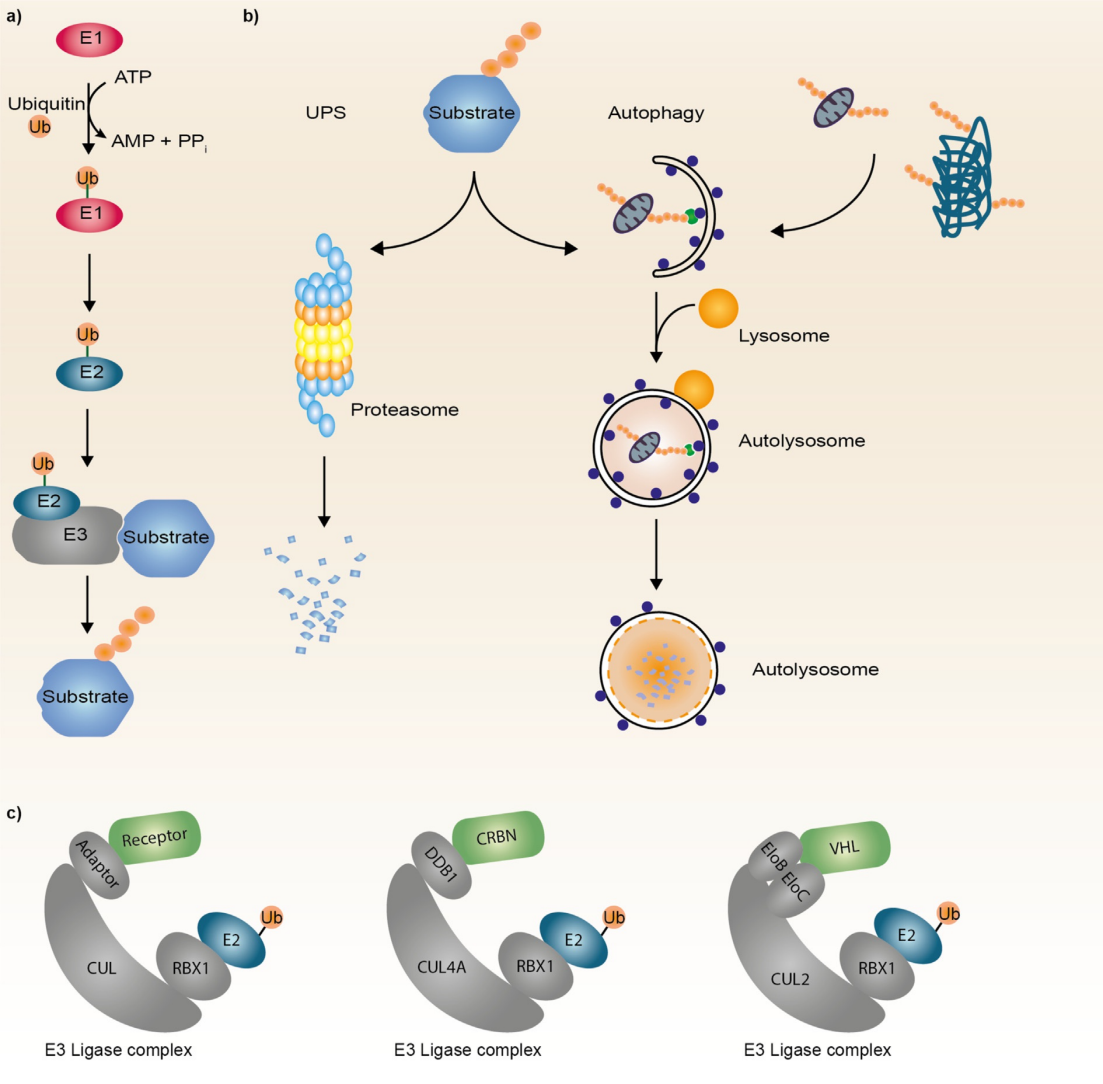
Native degradation pathways: a) Substrate ubiquitination occurs via a cascade of activation and transfer reactions mediated by the E1, E2 and E3 ubiquitin enzymes. b) Polyubiquitinated proteins are recognized and degraded by the proteasome. Organelles and protein aggregates are removed via autophagy, whereas a double membrane structure encapsulates the substrate and forms the autophagosome. Upon fusion with a lysosome the autophagosome becomes an autolysosome initiating the degradation process. c) Schematic representation of different Cullin RING ubiquitin ligases (CRLs). CRLs consist of a core protein Cullin (CUL), which is regulated by neddylation, and the RING protein ligase RBX1, interacting with the E2 conjugating enzyme. Substrate specificity is achieved via adaptors and substrate receptors such as Cereblon (CRBN) or the von Hippel‐Lindau protein (VHL) as well as the E2 ligases. Ub=Ubiquitin.

Native protein degradation occurs via one of two main mechanisms in the cell: the ubiquitin–proteasome system (UPS) and the autophagy‐lysosome pathway (Figure 1). Proteins are marked for degradation by a covalent post‐translational modification with the 76 amino acid protein ubiquitin.^7^ Covalent attachment of ubiquitin to the substrate follows a three‐step mechanism involving E1, E2 and E3 enzymes (Figure 1 a).^8^ Subsequent sequential ubiquitination of the conjugated ubiquitin results in the formation of a polyubiquitin chain. Ubiquitin contains seven lysine (Lys) residues for polyubiquitin chain formation (Lys 6, Lys 11, Lys 27, Lys 29, Lys 33, Lys 48, or Lys 63). While Lys 48 and Lys 11 linkages mediate proteasomal degradation, Lys 63‐linked ubiquitin chains are prone to degradation via autophagy.^9^

While the 26S proteasome efficiently degrades short‐lived, soluble unfolded or misfolded proteins and polypeptides,^10^ the autophagy–lysosome system is responsible for elimination of long‐lived proteins, insoluble protein aggregates and dysfunctional organelles such as degenerated mitochondria.^11^ There are three main forms of autophagy: macroautophagy, microautophagy and chaperone‐mediated autophagy.^12^ In this review, the focus is on macroautophagy (here referred to as autophagy). Autophagy is characterized by the formation of a double‐membrane structure, termed autophagosome, which sequesters cytosolic proteins, protein aggregates or dysfunctional organelles (Figure 1 b).^13^ These autophagosomes subsequently fuse either with endosomes or directly with lysosomes to form autolysosomes, ultimately resulting in the destruction of their contents by hydrolytic enzymes.^12^

Ubiquitination is at the heart of the degradation machinery for proteasomal‐ as well as autophagy‐mediated degradation, with E3 ligases as the critical components of the ubiquitination cascade. E3 ligases provide strict spatial and temporal substrate specificity and over 600 genes encoding for ubiquitin E3 ligases in the human genome, contributing to the cellular specificity and complexity of the ubiquitination system.^14^ E3s have been structurally classified in really interesting new gene (RING), Cullin‐RING‐, U‐Box‐ and homologous to the E6‐AP carboxyl terminus (HECT)‐type E3 ligases and each class employs a different mechanism for ubiquitin conjugation.^15^ The largest superfamily of E3 ligases, the Cullin‐RING E3 ligases, are multi‐subunit modular complexes which simultaneously bind the target protein and the E2 enzyme, enabling the transfer of ubiquitin from the E2 protein to the target. They consist of four main components, a Cullin protein serving as a scaffold protein, a RING finger protein which can bind to the E2 enzyme, a substrate receptor recognizing the target and an adaptor protein bridging the receptor with the Cullin protein (Figure 1 c). HECT E3 ligases receive the ubiquitin on an active site cysteine residue from the E2 enzyme before transferring it to their target proteins. Only about 1 % of all E3 ligases have been explored in TPD to date with Cullin‐RING E3 Ligases being the main E3s exploited so far. Predominantly, the E3 ligase receptor proteins Cereblon (CRBN) and von Hippel Lindau (VHL) are utilized for TPD followed by the inhibitor of apoptosis protein (IAP) and the E3 ligase mouse double minute 2 (MDM2). Increasing the knowledge of structural properties and expression pattern as well as the repertoire of E3 ligase binders bears the potential to expand the application of E3 ligases in therapeutic drug discovery, particularly TPD.

This review provides a comprehensive overview of various TPD techniques for chemical biology and drug discovery, highlighting their strengths and limitations from a medicinal chemist's perspective. To discriminate between the different methods, we divided them into two main categories: 1. Degradation of tagged target proteins; 2. Degradation of untagged target proteins. A protein tag comprises a peptide sequence or protein genetically fused to the protein of interest (POI) to allow its selective detection and/or purification.^16^ In doing so, the tag allows highly selective recognition of the POI without the need to identify a suitable binder like a small molecule, peptide or antibody. While no suitable target protein binder needs to be identified using tagged proteins comes at the cost of modifying the genome of the target cell/organism in advance, limiting the degradation of tagged target proteins predominantly to the field of chemical biology and target validation. In contrast, the degradation of untagged proteins allows studying the target in its native state but is dependent on the identification of a suitable target binder. However, as it is independent of genetic alterations to the organism, it is universally applicable. Analog to a drug discovery process the introduction of tagged TPD tools are evaluated first (section 2), which allow target validation and target discovery. In the following the focus is on untagged TPD strategies (section 3), that are based on specific binders and hold great potential for future therapeutic applications.

## 2 Degradation of Tagged Target Proteins

To generate a better target understanding and validate a target in a disease context controlling protein activity and abundance is a very powerful tool. Although, methods like CRISPR/Cas^17^ have dramatically impacted the target identification and validation workflow, inducing the degradation of the target protein in a fast, controlled and selective manner provides additional benefits.5d, 18 At the target discovery and validation stage of a drug discovery program there is typically no suitable binder available which allows to aim for degradation of the endogenous protein. This problem can be eluded by fusing a protein tag to the target protein enabling selective recognition of the tagged protein. A variety of tags, ranging from short amino acid sequences to protein domains are available to be introduced at the N‐ or C‐terminus of the POI.^16^ While some tags solely allow selective addressing of the target protein, others are directly inducing its degradation.^19^ In order to minimally perturb the system in question the protein tag should be endogenously inserted into the host genome using for example, CRISPR/Cas. Alternative methods, such as transfection/transduction of a DNA‐encoding plasmid in an either transient or stable fashion, might be considered as well but must be handled with care as they produce overexpressed protein levels which represent deviations from the native system. Additionally, in cases where protein overexpression is used the endogenous native protein levels are still present in the cell and can provide a constant basal activity.

### 2.1 Degrons

Maintaining protein homeostasis is pivotal for cell survival and proliferation. Therefore, the equilibrium between protein synthesis and degradation must be carefully regulated to keep the system in balance. Proteins, which are naturally degraded by the UPS, are displaying specific degradation signals, termed degrons, which represent E3 ligase binding motifs.10b, 20 Degrons can range from a single amino acid to short peptide sequences or domains and are transferable between proteins. Hence, attaching a degron to any other protein induces its degradation. Several constitutive and conditional degrons, which can be activated by small molecules, light or temperature, have successfully been utilized to induce target degradation.^19^, 20b

#### 2.1.1 Temperature Sensitive Degrons

**Figure 2:**
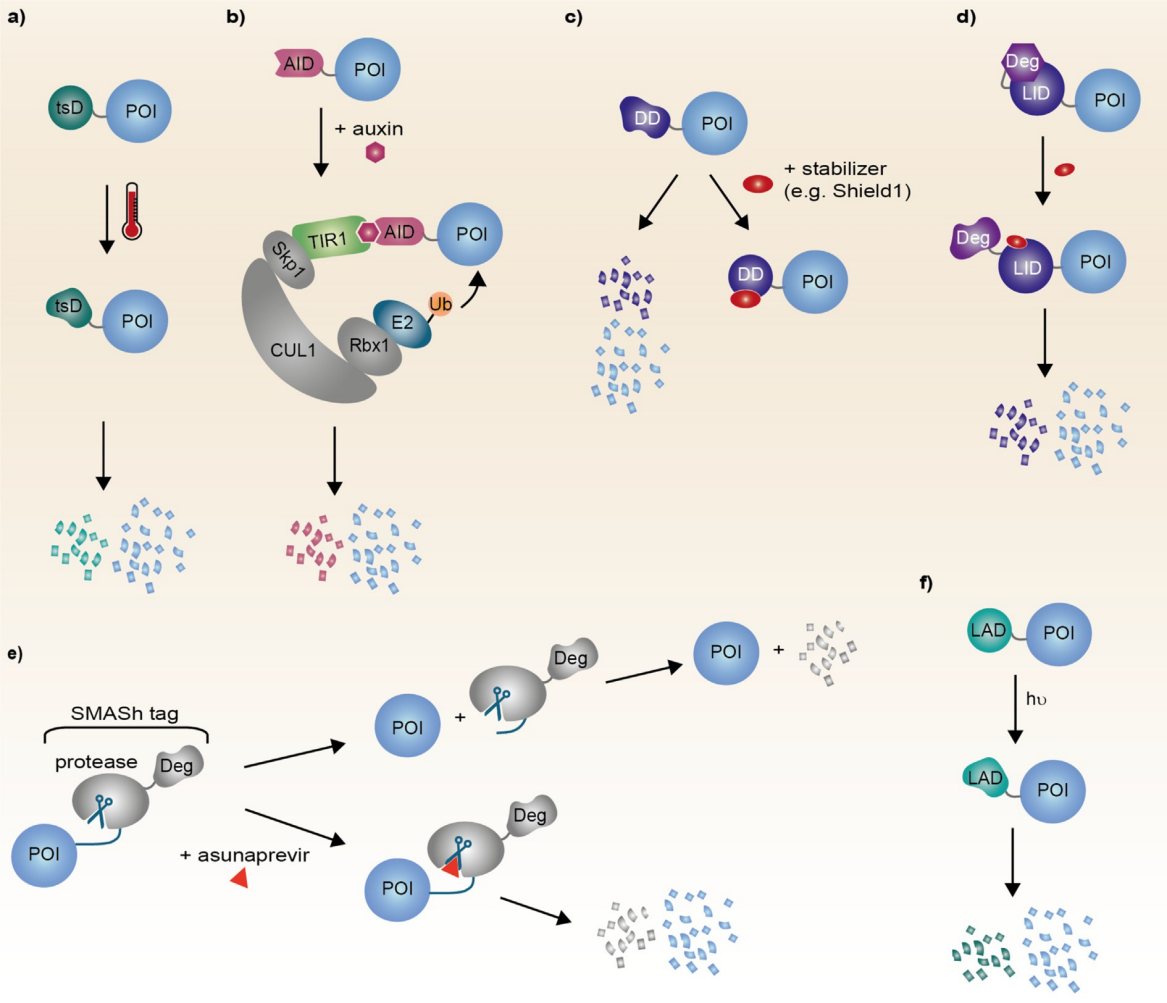
Summary of degron mediated degradation. a) Temperature sensitive degrons (tsD), b) Auxin‐inducible degron (AID), c) Destabilization Domain (DD) Degrons, d) Ligand‐induced degron (LID), e) Small molecule‐assisted shutoff (SMASh), f) Light‐activated degrons (LAD). POI=protein of interest; Deg=degron.

One of the first conditional degrons was identified in budding yeast and allows to induce temperature dependent protein degradation (Figure 2 a). This is achieved by fusing a temperature sensitive dihydrofolate reductase (ts‐DHFR, 25 kDa) to the target protein. The ts‐DHFR‐POI construct is stable at room temperature (23 °C–25 °C) but starts to unfold at physiological temperature (37 °C) inducing protein degradation.^21^ While this temperature‐mediated POI degradation is a powerful tool for budding yeast its use in other species is limited. A temperature difference affects the whole cell/organism and can lead to undesirable off‐target effects making them challenging to apply in higher order organisms.

#### 2.1.2 Small Molecule‐Induced Degrons

##### 2.1.2.1 Auxin‐Inducible Degron

A widely used and robust method to degrade a broad range of target proteins is the auxin‐inducible degron (AID). In plants, the hormone auxin triggers the degradation of the AID by means of the plant F‐box protein TIR1 and the CUL1‐RING ligase.^22^ Auxins, indole‐3‐acetic acid or 1‐naphthaleneacetic acid, bind to TIR1 in a molecular glue like fashion and recruit AIDs for degradation (Figure 2 b).^23^ As most proteins of the UPS are conserved in eukaryotes only TIR1 as well as the AID need to be ectopically introduced. Consequently, the AID system is bio‐orthogonal as neither TIR1 nor auxin are naturally present in mammalian cells.5d However, in some cases high concentrations of tryptophan or related metabolites can lead to some basal target degradation even in the absence of exogenous auxin. Thus far, the AID system has successfully been used to rapidly degrade both nuclear and cytoplasmic AID‐fused target proteins in various species as well as in human cell lines.^22^, ^24^ In order to reduce unwanted auxin‐independent TPD and to reduce the size of the AID (25 kDa) a much leaner version termed mini‐AID (7.4 kDa) was developed which can be efficiently introduced into the host genome using CRISPR/Cas.^25^ Despite the successful use in mammalian cells and the development of mini‐AID, the AID system is not currently amendable to use in vivo in mammals as auxin has to be used at very high concentrations. Consequently, this makes the design of a higher‐affinity AID very desirable.

##### 2.1.2.2 Destabilizing Domains

As mentioned above the AID demands, in addition to the AID itself, the exogenous introduction of the F‐box protein TIR1 to achieve degradation. An alternative presents the use of destabilizing domains (DDs) which constitutively induce the degradation of the target protein themselves. Upon ligand binding the DD is stabilized in a dose dependent manner thus preventing the degradation of the DD‐POI fusion construct (Figure 2 c). Thus far, DDs have been described for several protein/ligand combinations such as FKPB/Shield‐1 (rapamycin analog),^26^ DHFR/trimethoprim,^27^ UnaG/bilirubin^28^ or the human estrogen receptor binding domain/tamoxifen,^29^ respectively. A variety of species as well as diverse cellular proteins like kinases, cell cycle regulatory proteins, transcription factors and GTPases have successfully been addressed using DDs.^26^, ^30^ Maintaining a constant level of the rescuing agent to prevent degradation of the POI can be challenging. To reverse this process and to increase the flexibility of the DD system a conditional FKBP degron was designed which is degraded upon incubation with Shield‐1 instead of its absence (Figure 2 d).^31^ Unlike the previous described DDs the ligand‐induced degron (LID) can be fused to the C‐terminus of the target protein only.

##### 2.1.2.3 Small Molecule‐Assisted Shutoff

A highly interesting degron concept is described by small molecule‐assisted shutoff (SMASh) which belongs to the family of destabilizing tags. SMASh combines a constitutive degron with a protease and its corresponding cleavage site all derived from the hepatitis C virus (Figure 2 e).^32^ After expression of the SMASh‐POI fusion construct the SMASh degron (34 kDa) comprising the constitutive degron is auto‐cleaved from the target protein by the embedded protease. This results in the release of the non‐modified, native POI as well as the cleaved SMASh degron which is subsequently degraded due to its constitutive degron. Addition of the hepatitis C virus protease inhibitor asunaprevir^33^ inhibits the activity of the protease within the SMASh degron resulting in degradation of the whole SMASh‐POI fusion construct. Like many previously described degrons the SMASh system can be either fused to the N‐ or C‐terminus of the target protein and has been studied in mammalian cells as well as yeast.^32^ Introducing the SMASh‐tag to the host genome via CRISPR/Cas creates the endogenous protein (without asunaprevir) or induces its degradation (addition of asunaprevir) in a dose dependent manner making SMASh a highly interesting strategy for target validation. As no existing target protein and only newly synthesized protein is degraded upon asunaprevir addition the SMASh systems can be used to study protein half‐life. The time required for depletion of the target protein solely depends on its native half‐life.

#### 2.1.3 Light‐Activated Degrons

Activating a degron via temperature or a small molecule is a very convenient strategy to modulate protein abundance but does not provide a spatial component to control the target protein levels. To allow spatiotemporal control over the target light‐activated degrons (LADs) were designed (Figure 2 f). In a straightforward approach auxin was capped using a photo cleavable protecting group which can be removed after light irradiation.^34^ A more sophisticated approach describes the photosensitive degron (20 kDa), a truly light induced degron, which is independent of ligand binding.^35^ Illumination induces a conformational shift in the light sensitive domain exposing an unstructured region, which is degraded by a ubiquitin‐independent mechanism via the proteasome. A similar system, termed blue‐light‐inducible degron, has been used to rapidly (*t*_1/2_=30 min) degrade a target protein fusion construct in mammalian cells and zebrafish upon blue‐light irradiation.^36^ Controlling protein levels using light is an elegant and a marginally invasive method which holds great promise for further application.^37^ However, depending on the envisioned organism photosensitivity and light penetration might hamper the application of LADs.

### 2.2 Tag Specific Degraders

A very powerful tool to quickly achieve TPD for a wide range of target proteins and without the need to identify a specific binder represents the use of tag specific degraders. In this case, the degrader molecules are optimized to engage the protein tag resulting in effective removal of the tag‐POI fusion construct. The two main tags which are widely used as chemical biology tools for this technology are the HaloTag^38^ and the dTag system.^39^ Compared to many other techniques discussed in this manuscript the mode of action by which the bifunctional degraders designed to degrade HaloTag and FKBP^F36V^‐fusion (dTag system) proteins are working is well characterized and understood (for more detailed information please see section 3.3 PROTACs). In short, the bifunctional degrader brings the tagged protein as well as an E3 ubiquitin ligase in close spatial proximity inducing ubiquitination and subsequent proteasomal degradation of the tagged protein.^3^, 5a

**Figure 3:**
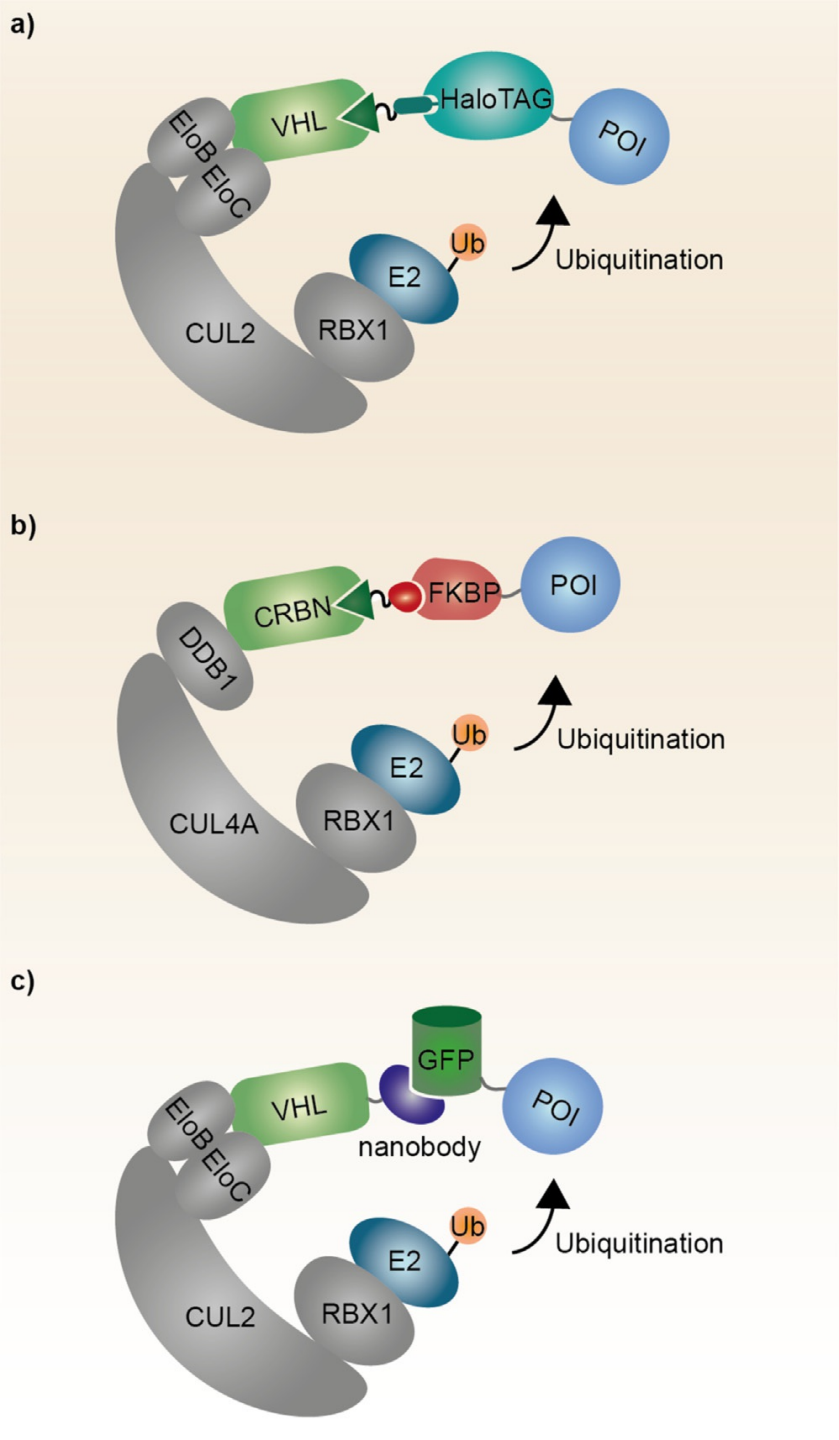
Schematic representation of the HaloTag, dTag and Nanobody approach. a) The HaloTag ligand covalently binds to the HaloTag and induces POI degradation via VHL. b) The dTag degrader brings the FKBP^F36V^‐tagged POI in close proximity to CRBN which subsequently ubiquitinates the fusion protein. c) The GFP nanobody fused to VHL recognizes the GFP‐POI fusion protein and mediates its removal via the UPS. POI=protein of interest; GFP=green fluorescent protein; Ub=ubiquitin.

The HaloTag (33 kDa) is a widely used self‐labeling tag introduced by Promega® which covalently binds to chloroalkanes.^40^ This allows highly selective covalent labeling of the HaloTag protein with a synthetic ligand of choice. Attaching an E3 ligase binder to the chloroalkane HaloTag ligands results in efficient and selective degradation of the target protein construct (Figure 3 a).^38^ HaloTag degraders have been designed to hijack VHL and IAP E3 ligases, thus far. They have been effective in the low nanomolar range to induce fusion protein degradation in overexpression models as well as in combination with endogenous CRISPR/Cas gene editing. HaloTag degraders have to be applied in stochiometric amounts due to their covalent nature and subsequent removal from the system together with the target protein. This is a general drawback for the HaloTag system as the catalytic turnover presents an attractive feature of PROTAC‐mediated protein degradation.

In contrast, applying sub‐stochiometric amounts of the bivalent degrader to achieve full target degradation can be achieved using the dTag system.^39^ The dTag approach comprises a FKBP^F36V^ protein tag (12 kDa) fused to the target protein as well as a bivalent degrader molecule (dTag). The dTag selectively binds to FKBP on one site and the E3 ligase Cereblon (CRBN) on the other site and thereby induces degradation of the fusion protein construct (Figure 3 b). The dTag approach has already been used multiple times for target validation in various disease models.^41^ In contrast to the HaloTag system the dTag can act catalytically and has proven to work for both, in vitro as well as in vivo applications.

### 2.3 Nanobody Induced Target Degradation

Another strategy to achieve selective TPD is based on genetically encoded nanobodies fused to E3 ligases, more specifically their substrate recognition proteins (Figure 3 c). Typically, this technology is used to degrade green fluorescent protein (GFP)‐POI fusion constructs using a GFP specific nanobody. Nanobodies are small single chain polypeptide antibodies (12–14 kDa) derived from camelid species and can be raised against different antigens. Initial studies were performed in *Drosphila* and *C. elegans* as well as mammalian cells.^42^ To establish a timely control over the system the expression of the nanobody‐VHL fusion can be achieved by using a tetracycline inducible vector system.42d Similar results can be obtained using an auxin‐dependent nanobody approach where the GFP‐targeting nanobody is fused to the AID.^43^ The genetically encoded degraders (described in section 3.5) resemble the next generation of the anti‐GFP nanobody approach. These degraders are not directed against a GFP fusion construct and are directly addressing the target protein itself, thus allowing for genetically encoded tag free induced degradation.

## 3 Degradation of Untagged Target Proteins

The advantage of targeting untagged proteins is that no genetic alterations are required to modify the system in advance. This setup mimics the pathological state more closely as no tag hampers the function, expression and availability of the target protein and allows to rapidly switch from one cell line to another as well as between organisms or species, respectively. Consequently, being able to degrade untagged target proteins is pivotal to move towards therapeutic applications. However, everything depends on the availability of a suitable target binder to engage the desired target protein or novel strategies to identify molecular glues.

### 3.1 Hydrophobic Tagging

**Figure 4:**
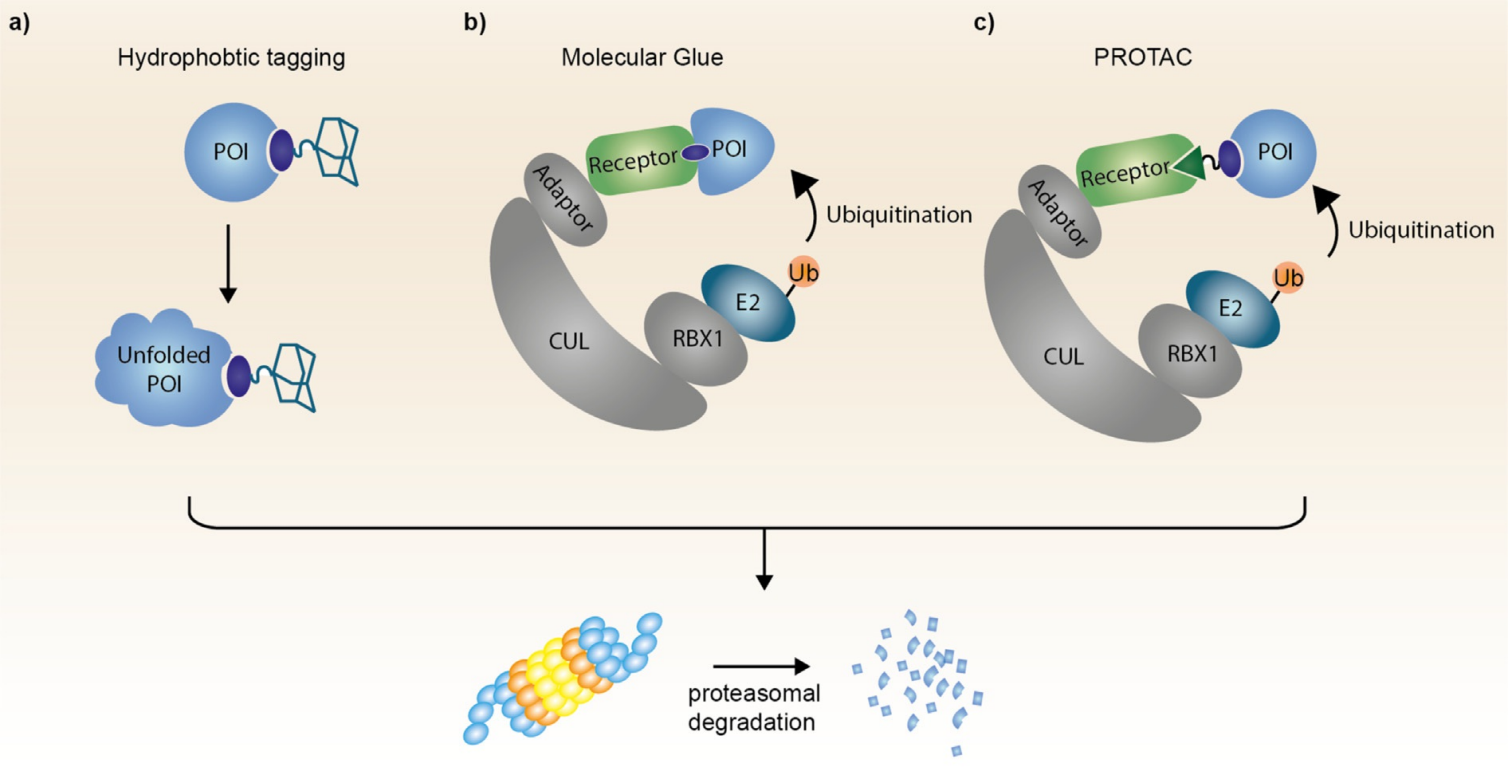
Schematic representation of hydrophobic tagging (HyT), molecular glue and PROTAC mode of action. a) The hydrophobic moiety of the HyT partially unfolds the POI resulting in chaperone recognition and subsequent proteasomal degradation. b) Upon binding of the molecular glue to the E3 receptor protein it reshapes the receptor's surface inducing the recognition of neo‐substrates. c) In contrast to molecular glues the PROTAC binds to both, the E3 receptor as well as the POI, bringing the POI in spatial proximity to the E3 and thus enabling its ubiquitination. POI=protein of interest; Ub=ubiquitin.

Maintaining protein homeostasis is essential for the overall wellbeing of the cell. Therefore, unfolded and misfolded proteins need to be immediately recognized and removed by the cell's quality control machinery. This occurs via a process called unfolded protein response (UPR).^44^ A characteristic feature of misfolded proteins is the exposure of hydrophobic residues or unstructured domains to the solvent, which is recognized by molecular chaperones. Chaperones either rescue the misfolded proteins by supporting their refolding or mediate their degradation by the proteasome in cases refolding is not successful.^45^ A method termed hydrophobic tagging (HyT) takes advantage of the UPR machinery by exposing hydrophobic patches on the surface of target proteins thereby triggering their degradation.^46^ An adamantyl group or Boc_3_Arg is covalently attached to a target binder mimicking the partially unfolded protein domain which can induce UPR upon ligand binding (Figure 4 a).^3^ Although HyT has successfully been used to degrade several target proteins such as the androgen receptor or the pseudokinase Her3,^47^ it is predominantly applied as a tool in chemical biology to study UPR in different cellular compartments or to degrade HaloTag fusion proteins.^48^ In general HyT suffers from low bioavailability and often shows incomplete target degradation. However, Faslodex (AstraZeneca®), a selective estrogen receptor degrader, is approved for the treatment of hormone‐receptor positive breast cancer.^49^ Nevertheless, it is important that Faslodex was not designed as a HyT and it is not clear to which extend its therapeutic benefit depends on the induced target degradation.

**Figure 5:**
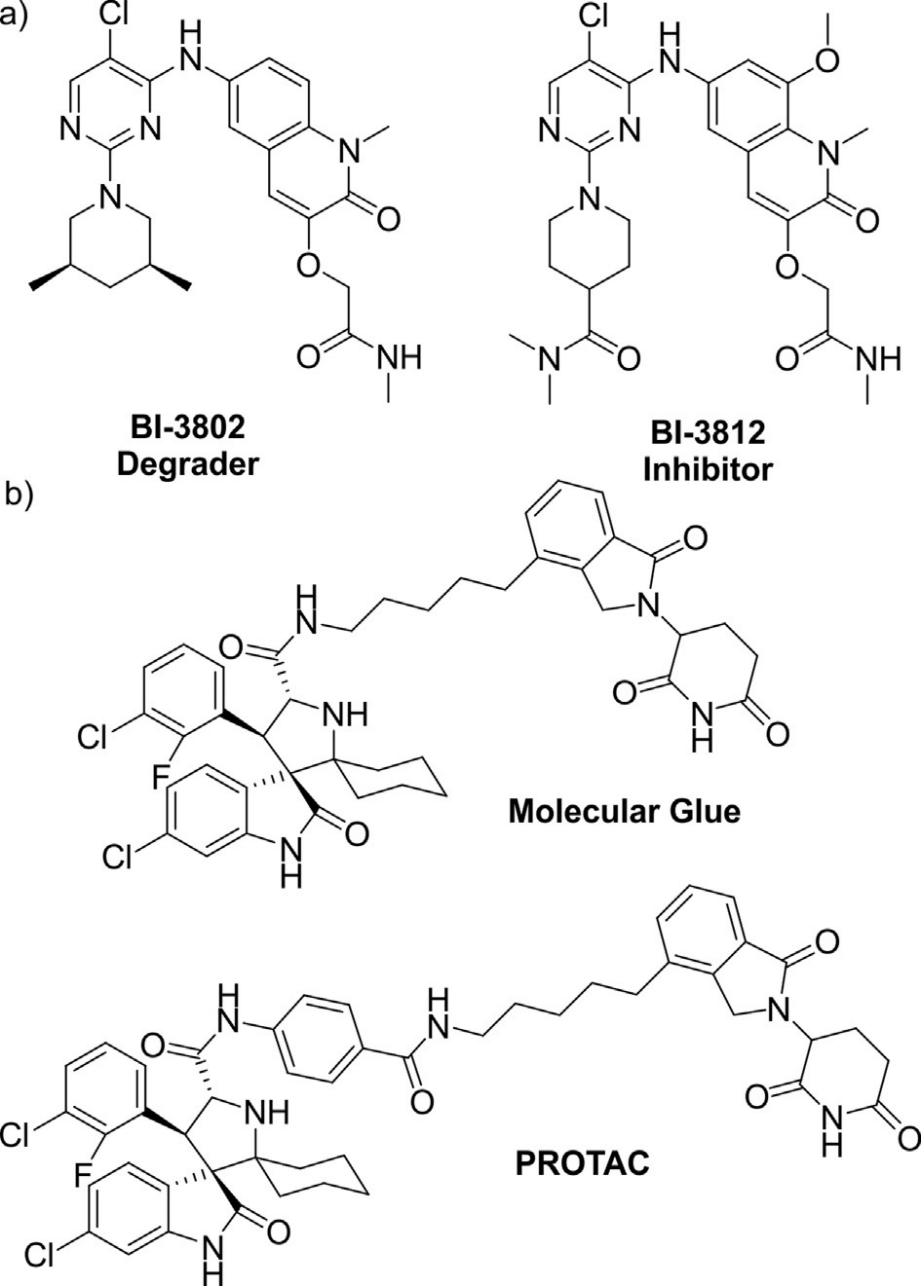
Structures of protein degrader. a) Structures of the BCL6 degrader **BI‐3802** and its close analog **BI‐3812** a BCL‐6 inhibitor. b) Structures of the GSPT1 degrading molecular glue and the mouse double minute 2 (MDM2) degrading PROTAC.

Sometimes the structural modification turning an inhibitor into a degrader can be very subtle. For example, **BI‐3802** induces the degradation of BCL6 in a proteasome dependent manner and significantly outperforms the corresponding inhibitor **BI‐3812**, a close analog (Figure 5 a).^50^ The exact mode of action for **BI‐3802** still remains elusive but it does not carry a large hydrophobic moiety like the typical HyTs. This poses the questions of how many other supposedly inhibitors are out there that act via protein degradation.

### 3.2 Molecular Glue

Hijacking and reprogramming E3 ligases to induce novel protein‐protein interactions (PPIs) and the degradation of neo‐substrates—non‐native substrates recruited by exogenous ligands—is a method already used and optimized by many different viruses to defend themselves against the host's response.^51^ However, it was still surprising to identify a small molecule, thalidomide, which is able to modulate the substrate scope of the E3 ligase CRBN upon binding.^52^ Thalidomide and its close analogs pomalidomide and lenalidomide are part of the immunomodulatory imide drugs (IMiDs) which reprogram CRBN to degrade several zinc finger transcription factors.6b, 53 IMiDs and other monovalent E3 ligase binders which are able to induce neo‐substrate degradation are often referred to as molecular glues (Figure 4 b).6c, 54 They induce the interaction of two proteins which otherwise do not show a native affinity for each other. However, with the current understanding the design of molecular glues is still hinging on serendipitous discoveries and only very few systems beside the CRBN/IMiD system have been identified thus far.^55^ Surprisingly and without intention, while trying to optimize a CRBN based MDM2 bifunctional degrader, small structural variations of the molecule lead to the identification of a molecular glue selectively degrading the translation termination factor GSPT1 which has been identified as an relevant off target of heterobifunctional phthalimid based degraders (Figure 5 b).^56^ Due to their small molecule nature and corresponding drug‐like properties molecular glues are highly interesting for therapeutic applications in the field of TPD.6a, 6b

### 3.3 PROTACs

The technique currently creating by far the most excitement in the field of TPD are PROTACs. Compared to other approaches like TRIM‐away (section 3.4) or hydrophobic tagging (section 3.1) PROTACs are highly modular. As large small‐molecules they show borderline drug‐like properties but have surprisingly proven suitable for oral application.^57^ Among all TPD approaches PROTACs are the most rational as ligands for the target protein and the E3 ligase are specifically selected. However, everything depends on the availability of a suitable target binder. Highly potent and efficacious PROTACs have since been developed against a variety of target proteins such as the bromodomain BRD4, the androgen receptor or Bruton's tyrosine kinase.^58^ Based on the tremendous success of PROTAC‐mediated protein degradation, the first two PROTACs developed by Arvinas®, ARV‐110 and ARV‐471, have entered clinical trials in 2019 targeting the androgen receptor and the estrogen receptor, respectively. First results published by Arvinas® describing human PK data reveal human exposure in the preclinical efficacious range with long half‐lives of 24 h up to multiple days.^59^ Initial clinical efficacy data has been presented at the ASCO 2020 annual meeting confirming the PROTAC mode of action in humans. As the focus of this review is a general overview of various chemical biology techniques to induce TPD more detailed literature on PROTACs can be found elsewhere.^3^, ^5^, ^60^

**Figure 6:**
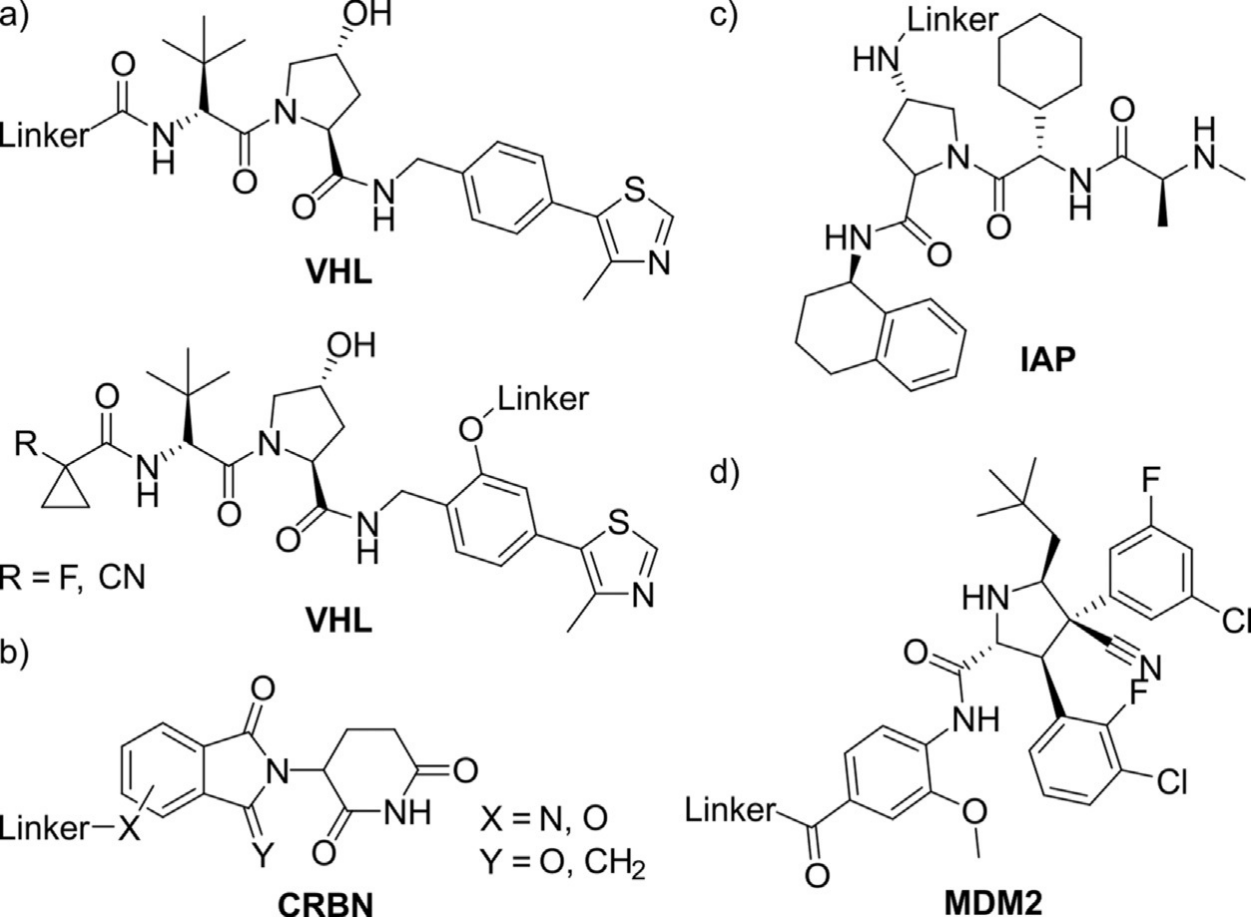
Structures of typical E3 ligase binders. a) The VHL binding peptidomimetics are based on the essential hydroxyproline. The linker for PROTAC synthesis can be either attached at the N‐terminus or side‐on (e.g. at the phenyl ring) to the peptidomimetic VHL binder. b) CRBN ligands are typically based on the IMiD scaffolds of thalidomide, pomalidomide and lenalidomide. c) IAP binders comprise peptidomimetics bearing a terminal N‐methylated alanine which is essential for binding. d) For MDM2 PROTACs idasanutlin is a commonly used MDM2 ligand.

PROTACs are bifunctional molecules comprising a target binding moiety, an E3 ligase recruiter and a linker (Figure 4 c). The predominantly used E3 ligases for PROTAC‐mediated protein degradation are VHL and CRBN with fewer examples harnessing IAP or MDM2. Examples of typical E3 ligase recruiters are shown in Figure 6. PROTACs induce spatial proximity between the target protein and an E3 ligase via the formation of a ternary complex thereby inducing the ubiquitination of the POI. Subsequently, the ubiquitinated target protein is recognized and degraded by the proteasome. This event‐driven mode of action allows PROTACs to address functions out of reach for traditional small molecule inhibitors and expands the druggable space as biological function can be connected solely to target engagement and is not anymore depending on high affinity target inhibition.1a, 1b, 3 However, by engaging the native intracellular protein degradation machinery PROTACs are limited to intracellular target proteins. The PROTAC‐induced absence of a protein opens up the target scope to traditionally hard to address non‐enzymatic protein families like pseudo kinases or proteins acting as adaptors or scaffolds.^61^ Additionally, certain resistance mechanisms can be attenuated or circumvented via degradation of the target protein.58a, 58c Whereas already a 10‐fold loss in potency due to mutations of the target protein usually results in insufficient target inhibition (below IC_90_ or IC_95_) for small molecules, the residual binding affinity in combination with the subsequent target degradation renders PROTACs still efficacious making them less liable to many frequently observed resistance mutations.58h, 62 Nevertheless, cancer cells will still find a way to defend themselves against PROTACs. Several recently published studies have identified mutations of the E3 ligase complex and the signalosome complex as potential resistance mechanisms against different BRD4 degraders.^63^ It appears, that the acquired mutations in the UPS are either selective to the VHL or CRBN E3 ligase leaving the cell sensitive to the degrader engaging the respective other E3 ligase.

While empiric guidelines like the rule‐of‐five^64^ for the optimization of traditional orally available small molecules inhibitors have been developed and refined over the past decades, optimizing the bioavailability for large molecules like PROTAC is still in its infancy.^65^ It appears that many predictive assays used for small molecules are less predictive when it comes to PROTAC optimization. On the other hand, due to its catalytic mode of action already a low concentration of PROTAC can be sufficient for effective degradation. Half maximal degradation concentrations (DC_50_s) in the low‐ and sub nanomolar range are commonly observed even when binding to the E3 ligase or the target protein are only in the micromolar range.58d, 66 However, membrane permeability poses a significant challenge for molecules of this size and polarity. In a recent study analyzing cell permeability the investigated PROTAC showed a drastically reduced cellular uptake compared to its parent inhibitor.^67^ Despite the reduced uptake, the PROTAC was still able to degrade its target protein with nanomolar potency highlighting the PROTACs catalytic mode of action.^67^, ^68^ The ability to perform multiple cycles and act in substoichiometric amounts describes a key feature of the PROTACs technology making the use of covalent target binders very challenging.^69^ However, successful covalent Ras^G12C^ and BTK PROTACs have been identified, recently.^70^ Despite the lack of catalytic turnover covalent PROTACs might still prove valuable when addressing highly challenging targets. Surprisingly, the use of reversible covalent warheads seems also to have a favorable impact on the cellular uptake of certain PROTACs when targeting a particular cysteine.70b, 71 Chemoproteomic analysis of many PROTACs revealed their generally excellent selectivity.61b, 68, 72 Furthermore, starting from a promiscuous kinase inhibitor such as foretinib, degraders with significantly improved selectivity profile can be obtained.66b, 72b

**Figure 7:**
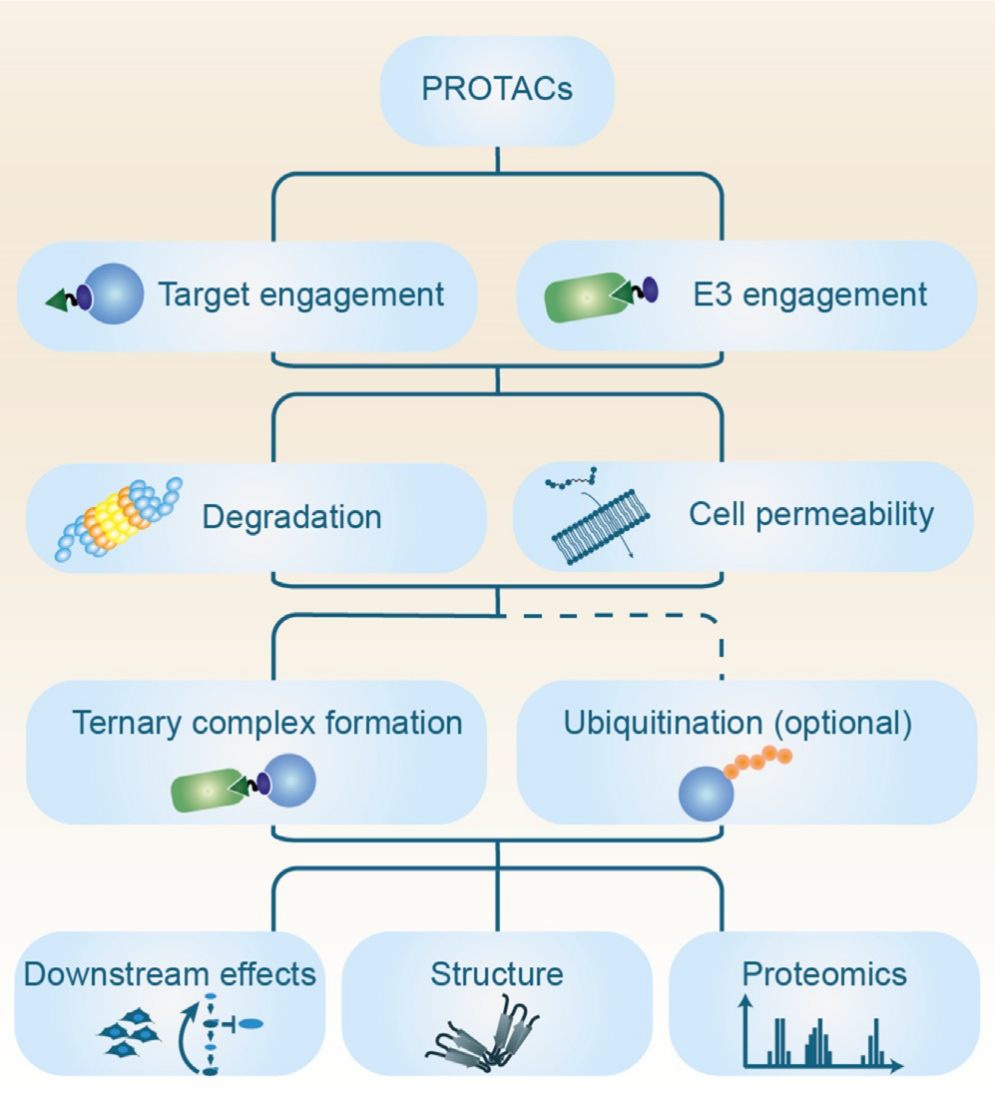
Representative PROTAC screening tree.

Optimizing a beyond rule‐of‐five compound with a molecular weight most likely between 700 and 1200 g mol^−1^ for clinical applications is an enormous challenge as many principles established for small molecule inhibitors do not translate to larger molecules. Despite these challenges, Arvinas® has managed to identify orally available PROTACs for their first two clinical programs and other companies claim to have achieved oral bioavailability for their PROTACs, as well. With only very few design guidelines available PROTAC optimization is still no straight forward process.65b This makes a carefully assembled screening platform invaluable for the characterization of the vast number of PROTACs synthesized during an optimization campaign. A representative screening tree is outlined in Figure 7. While some assays are straight forward to set up and can be run in an HTS format other assays need to be tediously optimized or are limited by low throughput. To degrade a target protein a PROTAC must successfully accomplish a cascade of different steps: i) enter the cell ii) engage the target protein and the E3 ligase iii) form an active ternary complex with the target protein and the E3 ligase iv) ubiquitinate the target protein v) degrade the target protein.^73^ While all these processes can be interrogated in individual assays not all of them are adding any value when you are looking to design your first PROTAC for a POI. From our perspective, the most important and feasible assays are biochemical and cellular target protein as well as E3 ligase engagement, and most of all target degradation. Consequently, in an industrial setting these assays need to be available in a high throughput format to be able to assess several hundred or more PROTACs. As most PROTAC programs so far are based on established small molecule targets, a target protein assay (inhibition or binding) is usually available or can be set up easily. The same is true for the most commonly used E3 ligases such as von VHL, CRBN, MDM2 or IAP for which different assays to monitor target engagement are commercially available.^74^ Comparing target engagement in live cells with in vitro or lysed conditions allows an evaluation of cellular uptake. Several published degraders have been evaluated in these assays and can be used as references. However, the most important challenge is to develop a suitable high throughput assay monitoring target protein degradation. While western blotting (WB) is the method of choice used to determine protein levels in almost all PROTAC publications the throughput is too low to evaluate a large number of molecules at various time points. Nevertheless, WB in a dose dependent manner should always be performed as a secondary assay to verify degradation as it is the most specific method using unmodified endogenous protein levels. As a high throughput compatible degradation assay several options are amendable with each assay offering opportunities and pitfalls. Using an overexpressed and tagged construct of the target proteins allows easy monitoring (e.g. GFP tagged proteins), quick setup and time resolved degradation in a high throughput format but due to potential high expression levels weak degraders might be missed or false positive PROTACs identified which only initiate degradation of the tag instead of the target protein itself.^75^ CRISPR/Cas modifications of the endogenous target protein adding a small tag (HA‐tag or NanoLuc®) are more time consuming but can increase sensitivity due to endogenous protein levels and a more favorable target protein/E3 ligase ratio. Immunofluorescence imaging is a valuable alternative to detect protein degradation but relies on the availability of a specific antibody and requires a constant supply of consumables. In cell western, TR‐FRET or AlphaLISA can be considered as well. To our knowledge a general strategy for monitoring high throughput protein degradation has not yet manifested with methods varying for each target protein depending on available tools. Nevertheless, hits from the high throughput degradation assays need to be validated in an orthogonal assay such as WB or capillary electrophoresis. For a full characterization of the degrader's downstream effects thorough proteomic analysis is inevitable. In this regard, chemoproteomics are emerging as the new workhorse and method of choice to characterize protein degraders as an unbiased approach is important. As has been shown previously, even weak binders can yield efficacious degraders and especially for CRBN based PROTACs all effected proteins need to be identified in order to monitor off‐targets and mitigate safety concerns.66b, 76

In case no active PROTACs can be identified using this setup or as additional support for rational PROTAC optimization further assays are useful. Especially monitoring the formation of the ternary complex comprising the target protein, the PROTAC and the E3 ligase is important at this point. Measuring ternary complex formation has been mainly done by using biophysical assays such as isothermal titration calorimetry (ITC) or surface plasmon resonance (SPR) which are both limited by low or medium throughput, respectively.^77^ On the other hand, the information obtained from these measurements is highly valuable and allows to assess cooperative binding, a key feature for many highly active PROTACs.77a Biochemical assays like time‐resolved fluorescence resonance energy transfer (TR‐TRET) or AlphaLISA can be used as well to assess ternary complex formation and provide a higher throughput compared to ITC or SPR.72a, 77c If ternary complex formation is evident, ubiquitination of the target protein can be checked. Suitable assays are provided by Promega® or can be run via tandem ubiquitin binding entity (TUBE) precipitation^78^ followed by WB detection of the target protein. However, these assays are usually only run in cases where no degradation can be observed and the PROTAC needs to be carefully characterized. If a working PROTAC is identified and degradation via the proteasome verified (addition of proteasome inhibitor as control) a ubiquitination assay becomes redundant.

To aid PROTAC optimization, attempts to crystallize the ternary complex in order to understand the orientation of the two ligands as well as the linker is highly recommended. Structural insight can be tremendously helpful to guide PROTAC design and can lead to surprising but useful tweaks not obvious at first glance.61b, 77a, 79 Computational modeling can give additional guidance when it comes to PROTAC and linker design and should be applied as early as possible during optimization. However, without structural guidance de novo prediction of the ternary complex is highly challenging due to the myriad of possible conformations. While initial PROTAC linkers usually comprise polyethylene glycol or alkyl chains to allow sufficient flexibility for ternary complex formation, the long‐term goal is to dial in rigidity in order to increase molecular recognition and overall stability. This process has been nicely demonstrated for the SMARCA2/4 PROTACs.61b

While several assays have been described for the in vitro optimization of PROTACs very little is known about how to design PROTACs for in vivo applications. Despite various xenograft studies using PROTACs are reported, optimization strategies for in vivo experiments are only beginning to emerge.58c, 58d, 80 The recently published study on improving bioavailability of a previously unstable Bruton's tyrosine kinase PROTAC to yield a compound with reduced clearance, increased half‐life and exposure is the beginning of hopefully many more examples to follow.^81^ Thus far, judging from a limited amount of data, it appears to be very challenging to establish an in vitro/ in vivo correlation for PROTACs. Without reliable prediction tools available, many PROTACs will need to be profiled in vivo to optimize bioavailability and efficacy in order to develop a clinical candidate.

#### 3.3.1 Light Controlled Targeted Protein Degradation

**Figure 8:**
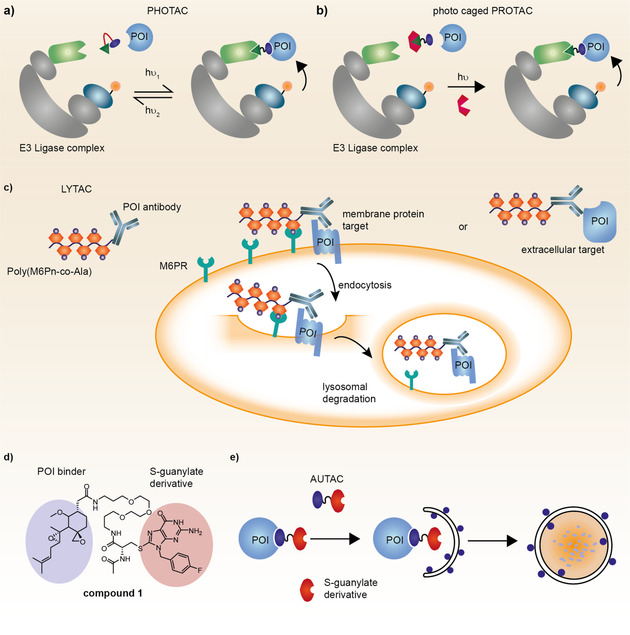
Schematic overview of PHOTACs, LYTACs and AUTACs. a) PHOTACs can be switched between an inactive and active conformation upon irradiation with different wavelength. b) Photo caged PROTACs are activated after removal of the photocleavable protecting group with light. c) LYTACs are degrading extracellular proteins by inducing endocytosis via the mannose‐6‐phosphate receptor (M6PR) and subsequent lysosomal degradation. d) Compound **1** induces the degradation of MetAP2 (POI) by means of autophagy. e) Schematic representation of AUTAC mode of action. POI=protein of interest.

Despite the excitement about PROTACs as a novel therapeutic modality, PROTACs bear also a huge potential as a chemical biology tool (e.g. section 2.2). The design of photo‐switchable PROTACs allows for temporal control of TPD (Figure 8 a).^82^ These PHOTACs82a or PhotoPROTACs82b can be switched between a biologically active and inactive state using different wavelength of visible light. In an additional approach to gain temporal control of protein degradation, photocaged PROTACs have been described (Figure 8 b).^83^ Upon irradiation with a certain wavelength the free PROTAC is released and degradation is induced. In further studies these light controlled PROTACs might be used to monitor TPD in distinct cellular compartments.

#### 3.3.2 Extracellular Targeted Protein Degradation

Complementary to PROTACs, lysosome targeting chimeras (LYTACs) allow the degradation of extracellular and membrane target proteins.^84^ A LYTAC is a bifunctional molecule comprising an antibody against an extracellular target protein which is fused to an agonistic glycopeptide ligand for the cation‐independent mannose‐6‐phosphate receptor (CI‐M6PR) (Figure 8 c). The CI‐M6PR internalizes upon ligand binding and transfers its cargo to lysosomal degradation while the receptor itself is recycled and shuttled back to the cell membrane. First proof‐of‐concept studies demonstrate the successful degradation of epidermal growth factor receptor (EGFR), programmed‐death‐ligand 1 (PD‐L1) and Apolipoprotein E4. However, at this point further studies are needed to assess the potential of this technology and any potential therapeutic application.

#### 3.3.3 Autophagy Mediated Targeted Protein Degradation

The second major pathway of protein degradation—autophagy—is pivotal for the degradation of misfolded proteins, aggregates and especially whole cell organelles. In a recent study the autophagy pathway has been hijacked for the first time to rationally degrade a desired protein as well as fragmented mitochondria (Figure 8 d).^85^ Based on an (S)‐guanylate derivative as a recognition tag for the autophagosome autophagy targeting chimeras (AUTACs) have been designed to remove cytosolic proteins and organelle targets. Due to their mode of action AUTACs will be limited to cytosolic targets and most likely not be able to act in a catalytic manner. In that sense, autophagy mediated TPD can be more precisely perceived as cargo loading onto the autophagosome and subsequent degradation than catalytic turnover as observed for molecular glues or PROTACs.

### 3.4 TRIM‐Away

**Figure 9:**
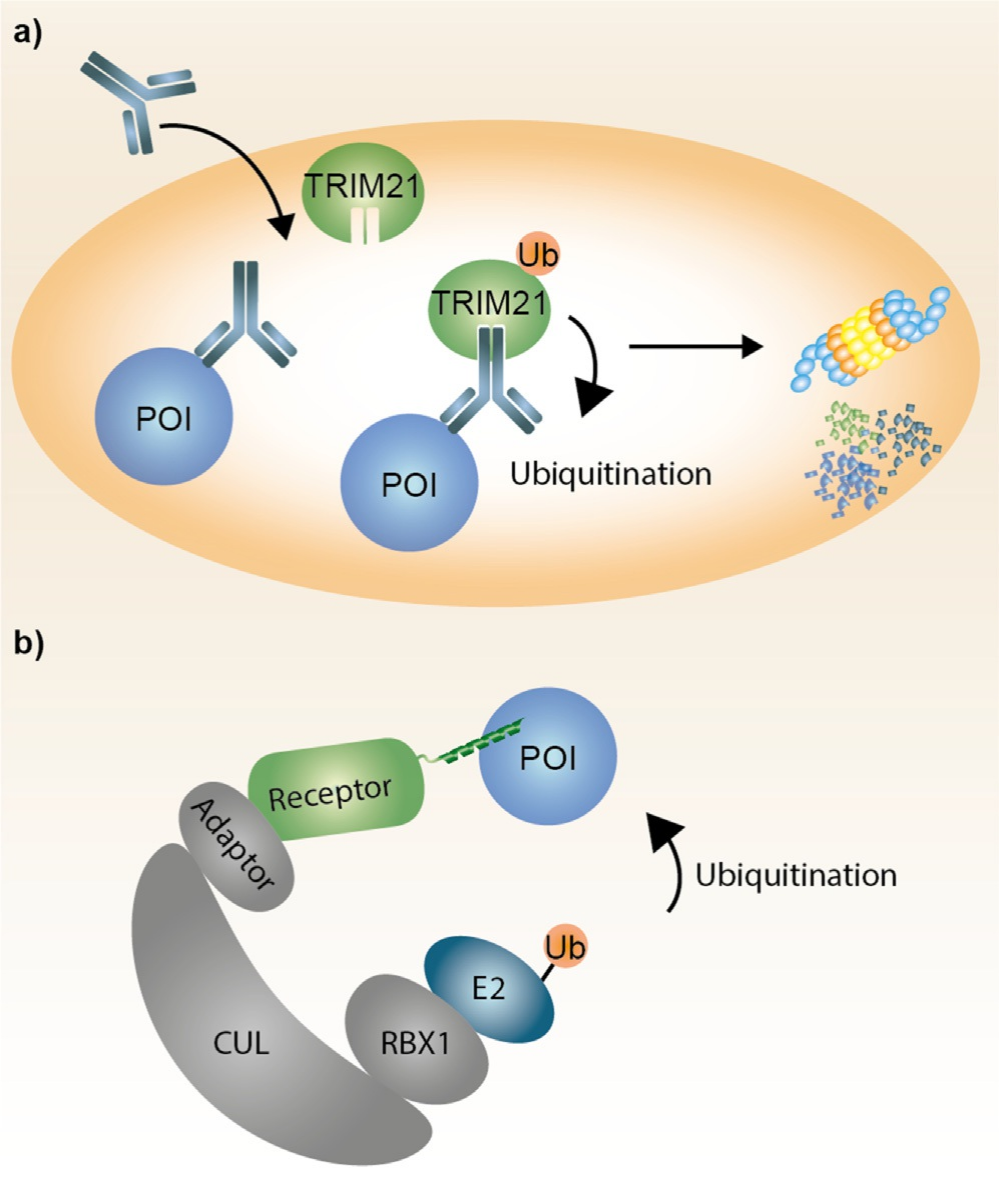
Schematic representation of TRIM‐Away and bioPROTACs. a) TRIM21 selectively recognizes the Fc‐region of an antibody which results in proteasomal degradation of the POI, the antibody as well as TRIM21. b) bioPROTACs are fused to the E3 receptor proteins and bind to the POI via a peptide or protein recognition domain. Subsequently, the POI gets ubiquitinated and degraded by the proteasome. POI=protein of interest; Ub=Ubiquitin.

In contrast to PROTACs which are currently investigated as potential remedies for diseases the TRIM‐away technology has a great potential to be a valuable tool in chemical biology and target validation.^86^ TRIM‐away harnesses the endogenous ubiquitin ligase TRIM21 which recognizes the constant Fc‐region of antibodies (Figure 9 a). Thus, proteins of interest can be targeted by a conventional antibody which upon TRIM21 recognition induces rapidly ubiquitination and degradation of the protein‐antibody complex via the proteasome. In contrast to the catalytic nature of PROTACs the whole complex of target protein, antibody as well as TRIM21 is degraded resulting in consumption of the antibody and the ubiquitin ligase. Consequently, endogenous TRIM21 levels might not be sufficient to achieve complete target degradation which demands TRIM21 supplementation via overexpression or external addition. For the successful application TRIM‐away requires a selective antibody against the POI which recognizes the native conformation of the target protein. Additionally, the antibody and supplemented TRIM21 must be delivered via microinjection or electroporation hampering its general applicability. TRIM‐away is particularly useful for target validation if DNA‐ or RNA‐based depletion methods have not been successful and can be also applied to non‐dividing primary cells for which conventional loss‐of‐function techniques are particularly challenging.

### 3.5 Genetically Encoded Degrader

As already mentioned above, the rational design of a targeted protein degrader relies on the availability of a suitable target binder. However, most proteins of the undruggable proteome are termed undruggable due to the lack of chemical matter. Biologics on the other hand can be raised against most protein targets but are limited to extracellular or surface proteins. Nevertheless, by fusing a peptide or antibody‐mimetic directly to a human or bacterial E3 ligase so called bioPROTACs were designed to specifically degrade challenging intracellular proteins (Figure 9 b).^87^ As only a few E3 ligase binders have been identified thus far, PROTACs are mainly harnessing the four above mentioned E3 ligases of the more than 600 available human E3 ligases. As bioPROTACs are engineered genetic constructs the whole repertoire of ubiquitin ligases can be utilized for target degradation. In a proof of concept study, the protein levels of the oncology target, proliferating cell nuclear antigen (PCNA), could be significantly reduced with 9 out of 16 tested E3 ligases and much faster than comparable RNAi‐based approached would allow.87a Additionally, nanobodies fused to several F‐box proteins were screened for degradation of activated RhoB(GTP) to study its cellular function.87d Although bioPROTACs can turn into powerful tools for interrogating the degradability of a substrate and guiding the selection of the best suited E3 ligase they will be most likely limited to chemical biology applications due to their dependence of genetic encoding.

## 4 Conclusion and Outlook

As highlighted by the various achievements within recent years, TPD has matured from a chemical biology niche to a fully established application for target validation and discovery. Furthermore, TPD in form of molecular glues is established on the market with billions of revenues created by the IMiDs alone and has even advanced to the clinic in the very young field of PROTAC‐mediated degradation in 2019.^57^, ^59^ Despite the tremendous success TPD is still in its infancy thus far only scratching the surface of its full potential especially with respect to expanding the druggable space. Piggybacking on the advances in genome editing technologies (e.g. CRISPR/Cas) as well as chemical proteomics TPD will further flourish as a powerful strategy in chemical biology foremost in the area of target validation and target mining. Within the near future we will see TPD develop as one of the standard procedures to verify hits identified via genome editing screens. With more TPD approaches already allowing an in vivo application target validation will raise to a completely new level.41b

With the myriad of methods to choose from one can easily be overwhelmed and struggle to find the best system for the problem at hand. For a condensed overview of the discussed techniques we summarized their key features in Table 1. However, judging from a medicinal chemist's perspective the most powerful chemical biology tools for target validation appear to be the SMASh‐tag and the dTag approach.^32^, ^39^ The SMASh‐tag requires only a single genetic modification of the host genome and either produces the native, untagged POI, as without addition of the inhibitor asunaprevir the active protease cleaves the degradation tag from the POI, or the SMASh‐POI fusion construct is degraded upon asunaprevir addition. Consequently, the studied system resembles the native state as closely as possible without a massive tag influencing the availability, activity or general properties of the target protein. Although, in vivo applications for the SMASh‐tag have thus far not been reported the small molecule degradation inducer asunaprevir is an approved drug and is predicted to be applied at dosages that are nontoxic in vivo.^32^ As a minor drawback, the SMASh‐tag can only control the fate of newly synthesized protein and has no effect on already expressed (and degron cleaved) protein levels. Therefore, the clearance of the POI from the system is solely dependent on the protein half‐life and might be not suitable for proteins with a very long half‐life. Despite the enormous potential of the SMASh‐tag technology only a few examples have been reported to date and it remains to be seen whether SMASh will gain broad acceptance. An alternative presents the dTag system which rapidly degrades the FKBP^F36V^‐POI fusion proteins from the system.^39^ Like the SMASh‐tag the dTag only requires a single genetic insertion and has already proven to be effective in vivo.41b, 88 However, the size and attachment to either N‐ or C‐terminus of the FKBP^F36V^‐tag (12 kDa) might influence the target protein in an unfavorable way and needs to be checked beforehand. Future advances in gene editing technologies will improve the ability to insert these tags on an endogenous level making them hopefully available to almost any desired cell line in the future.

**Table 1:** Summary of discussed TPD methods.

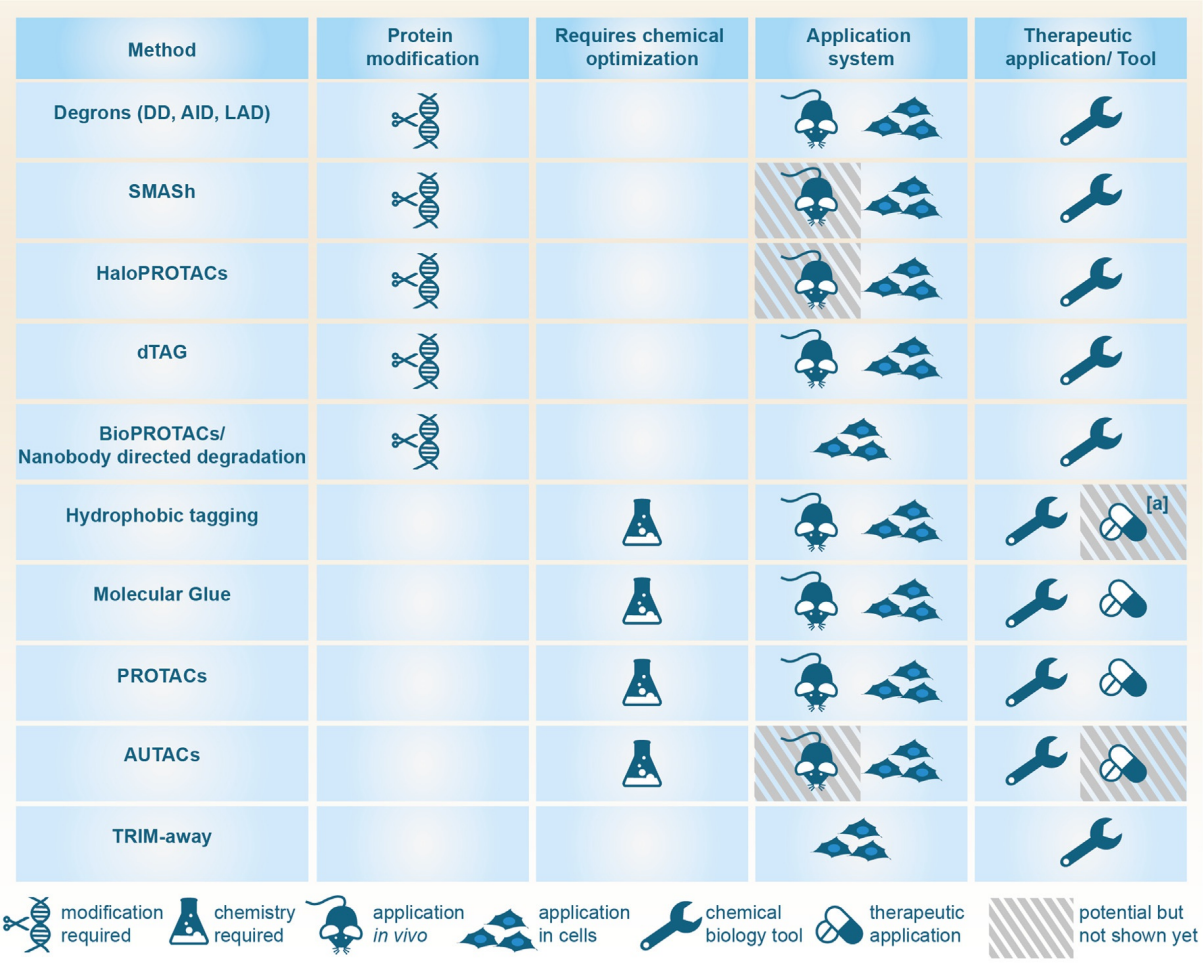

[a] Despite Faslodex being an approved drug it was not designed as an HyT.

While the outlook for the chemical biology side of TPD is highly encouraging it is equally bright for the therapeutic angle targeting untagged proteins. Already in the short period of rationally designing protein degraders, post elucidation of the IMiD mode of action (2010), several CRBN modulators with different selectivity profiles haven been identified and three are currently evaluated in clinical trials.6b, 89 This created an excitement towards the identification of additional molecular glue systems with the first results being reported recently.6a, 54b Besides molecular glues, PROTACs have entered the stage and rapidly progressed towards the first clinical trials.^57^, ^59^ PROTACs allow an even more rational approach to target almost any protein for degradation, provided a suitable binder can be identified. However, most PROTAC programs reported thus far are based on validated clinical targets. To reduce the risks when exploring a new clinical modality this cautious initial approach is reasonable but to unleash the full potential of PROTACs and expand the druggable space a new way of thinking in drug discovery must emerge. By inducing degradation of the target protein degraders can exhibit a differential biology compared to inhibitors and address scaffolding roles as well as enzymatic functions. This provides a chance as well as a pitfall for the early hit‐to‐lead process. In established hit‐to‐lead processes compounds are often excluded due to assumed off‐target effects without any follow up investigation if their cellular effects are stronger than their biochemical IC_50_s would predict. With respect to TPD, this process most likely has removed several potent protein degraders without ever realizing their existence. Consequently, sooner or later the hit‐to‐lead process in industry will have to be adapted accordingly. In the future, aiming for the identification of small‐molecule target protein degraders instead of inhibitors will emerge as a common strategy in early research.

Considering the recent successes of TPD and especially PROTACs experts in the field are excited to see whether PROTACs can live up to their high expectations. Although the first human PK data look promising PROTACs still have a long way to go until they will achieve approval and an even longer way to see how they will perform on the market. Crucial questions like: “How quickly will we see resistance emerge?” or: “What will the main resistance mechanism be?” are waiting to be answered. Efforts to pinpoint common PROTAC resistance mechanisms consistently found mutations hampering the UPS while leaving the target protein unscathed.^63^ Nevertheless, only long‐term clinical use will provide answers.

Despite these open questions the hunt to identify the next clinical candidates is in full swing. Almost every pharmaceutical company is pursuing TPD programs either alone or in collaboration with one of the young biotech companies in the field. Upcoming decisions on several patent applications are monitored closely by everyone working on TPD. While some companies are aiming to discover new chemical matter for the established E3 ligases others are focusing on unraveling the technology itself. However, a set of rules on how to design the perfect PROTAC for a given target remains to be crafted. Thus far, the correlation between in vitro and in vivo data still poses a massive challenge when it comes to PROTAC design. Additionally, immense efforts are underway to identify and hijack tissue or disease specific E3 ligases in order to address certain diseases more selectively or increase the therapeutic window. Without any doubt many things are happening around TPD and new ideas and directions are appearing on a constant basis. Inspired by the non‐native PPIs commonly observed in TPD, ideas are emerging to induce other post translational modifications besides ubiquitination. Early examples recruiting phosphatases or RNAses to eliminate proteins before they are produced will only be the tip of the iceberg.^90^

In light of the success of biologics and the emerging progress of nucleotide‐based therapeutics as well as the upcoming gene therapies, small molecule drug discovery has been looking for its own shot to address the undruggable proteome. TPD is among the technologies which helps to fill this void offering a path towards a medicinal chemistry based approach able to compete with many of the new modalities emerging within the last decades.^91^ Within the industry a goldrush‐like atmosphere developed, and it remains to be seen if TPD can live up to its high expectations. However, these are definitely great and exciting times to work on TPD and be able to actively shape the next generation of small molecule drug discovery.

## Conflict of interest

All authors are or were employees of Bayer AG.

## Biographical Information

*Laura Luh obtained her PhD working on transcription factors with implications in cancer at the Goethe University Frankfurt. After joining Evotec in Hamburg to establish new techniques to assess small molecule target interactions, she moved to Cambridge to join Anne Bertolotti's lab at the MRC Laboratory of Molecular Biology conducting her postdoctoral studies on specific phosphatase inhibitors in order to correct protein misfolding defects. In 2018, she joined Bayer to work on targeted protein degradation in different therapeutic areas*.

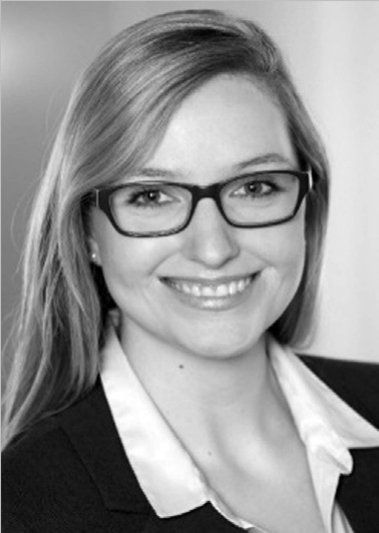

## Biographical Information

*Ulrike Scheib studied biochemistry in Tübingen and completed her master's thesis at the UC in San Diego. For her PhD in biophysics she joined the optogenetic research group of Peter Hegemann at the Humboldt‐University of Berlin in 2013 and studied rhodopsin‐guanylyl cyclase. In 2018 she started as a PostDoc at Bayer in Berlin and is particularly interested in structure–function relationships and protein degradation*.

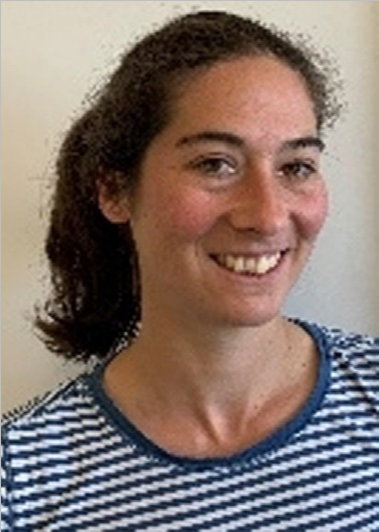

## Biographical Information

*Katrin Juenemann studied biology at the Friedrich‐Schiller‐University in Jena. She completed her PhD in Molecular biology at the Leibniz Institute on Aging in Jena and continued her scientific work on neurodegenerative diseases as a postdoctoral fellow at the Academic Medical Center in Amsterdam. In 2016 she joined the Leibniz Institute for Molecular Pharmacology as an AXA Research fellow to study intracellular ubiquitination and protein degradation associated with neurodegeneration. As a scientist in preclinical research at Bayer she works on targeted protein degradation*.

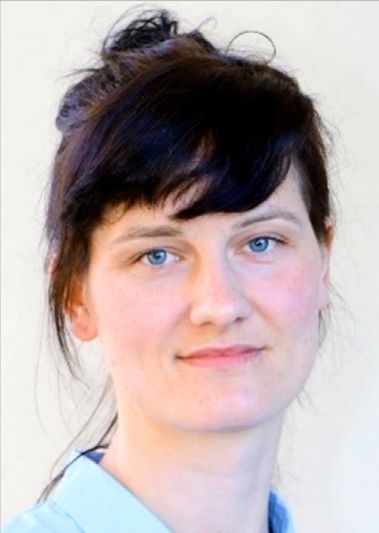

## Biographical Information

*Lars Wortmann studied chemistry at the University of Frankfurt. He received his Ph.D. from the RWTH Aachen and performed postdoctoral studies at Stanford University with a particular focus on stereoselective synthesis. In 2001 he joined the Medicinal Chemistry department of Schering AG (now Bayer AG). He has worked on several oncological projects and he is one of the main inventors of Bayer's clinical ATR inhibitor BAY 1895344. Additional interests: chemical probes for open science, PROTACs, chemical biology*.

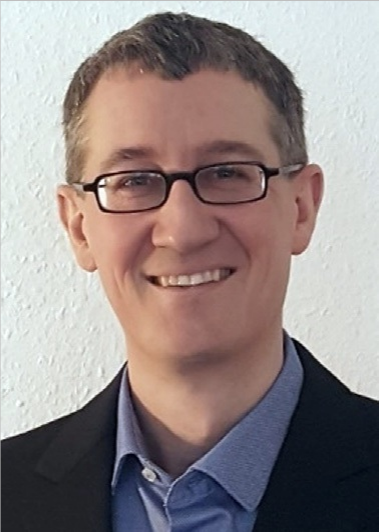

## Biographical Information

*Michael Brands studied chemistry in Münster and completed his doctoral thesis at the Max‐Planck‐Institut für Kohlenforschung in Mülheim in 1993. After a postdoctoral stay with Wolfgang Oppolzer at the University of Geneva, he started his industry career in Drug Discovery. Today he is head of the Small Molecule Innovation Department at Bayer Pharmaceuticals R&D responsible for Lead Discovery, Medicinal Chemistry and Thorium Conjugate Research. Since 2006, he teaches Medicinal Chemistry at the University of Paderborn*.

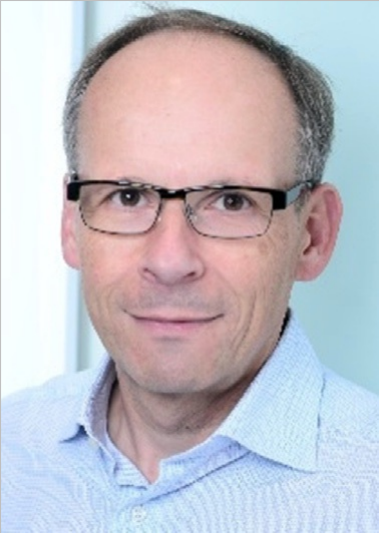

## Biographical Information

*Philipp M. Cromm studied chemistry at the Technical University Munich where he worked in the group of Horst Kessler. For his master's thesis he spent time with David J. Craik at the Institute for Molecular Bioscience and received his PhD under guidance of Herbert Waldmann at the Max‐Planck‐Institute for Molecular Physiology. Thereafter, he carried out postdoctoral studies in the group of Craig Crews at Yale University working on targeted protein degradation. In 2018 he joined Bayer AG as a Medicinal Chemist*.